# Outcomes of Adult Liver Retransplantation: A Canadian National Database Analysis

**DOI:** 10.1155/2022/9932631

**Published:** 2022-03-22

**Authors:** Peter D. Yoon, Madhukar S. Patel, Carla F. Murillo Perez, Tommy Ivanics, Marco P. A. W. Claasen, Hala Muaddi, David Wallace, Bettina Hansen, Gonzalo Sapisochin

**Affiliations:** ^1^Multi-Organ Transplant Program, Toronto General Hospital, Toronto, Ontario, Canada; ^2^Department of Surgery, Westmead Hospital, Sydney, Australia; ^3^Division of Surgical Transplantation, University of Texas Southwestern Medical Center, Dallas, TX, USA; ^4^Toronto Centre for Liver Disease, Toronto General Hospital, Toronto, Ontario, Canada; ^5^Department of Surgery, Henry Ford Hospital, Detroit, MI, USA; ^6^Department of Surgical Sciences, Akademiska Sjukhuset, Uppsala University, Uppsala, Sweden; ^7^Department of Surgery, Division of HPB & Transplant Surgery, Erasmus MC Transplant Institute, University Medical Centre Rotterdam, Rotterdam, Netherlands; ^8^Department of Health Services Research and Policy, London School of Hygiene and Tropical Medicine, London, UK; ^9^Institute of Liver Studies, King's College Hospital NHS Foundation Trust, London, UK; ^10^Institute of Health Policy, Management and Evaluation, University of Toronto, Toronto, Ontario, Canada

## Abstract

**Background:**

Liver retransplantation remains as the only treatment for graft failure. This investigation aims to assess the incidence, post‐transplant outcomes, and risk factors in liver retransplantation recipients in Canada.

**Materials and Methods:**

The Canadian Organ Replacement Register was used to obtain and analyse data on all adult liver retransplant recipients, matched donors, transplant-specific variables, and post‐transplant outcomes from January 2000 to December 2018.

**Results:**

377 (6.5%) patients underwent liver retransplantation. Autoimmune liver disease and hepatitis C virus (HCV) were the most common underlying diagnoses. Graft failure was 7.9% and 12.5%, and overall survival was 77.1% and 65.6% at 1 year and 5 years, respectively. In contrast to recipients receiving their first graft transplant, the retransplantation group had a significantly higher incidence of graft failure (*p* < 0.001) and lower overall survival (*p* < 0.001). The graft failure and patient survival rates were comparable between second transplant and repeat retransplant recipients. Furthermore, there were no differences in graft failure and patient survival when stratified according to time to retransplantation. Recipient and donor age (HR = 1.12, *p*=0.011; HR = 1.09, *p*=0.008), recipient HCV status (HR = 1.81, *p*=0.014), and donor cytomegalovirus status (HR = 4.10, *p*=0.006) were predictors of patient mortality.

**Conclusion:**

This analysis of liver retransplantation demonstrates that this is a safe treatment for early and late graft failure. Furthermore, even in patients requiring more than two grafts, similar outcomes to initial retransplantation can be achieved with careful selection.

## 1. Introduction

Liver retransplantation was first performed by Starzl et al. in 1968, with efforts being described to “have borne bitter fruit,” as only 6 of 27 (22%) patients survived past six months [1]. As retransplantation is the only treatment for irreversible graft failure after primary liver transplantation, further attempts demonstrated progress but with persistently increased mortality compared with primary transplantation [2–6]. Recent studies, however, have found no difference in survival between primary liver transplantation (LT) and liver retransplantation in appropriately selected recipients, partly due to the increase in sustained viral response with direct-acting antiviral agents (DAA) and the decrease in the number of patients undergoing repeat transplant for recurrent hepatitis C virus (HCV) [7].

Despite more contemporary reports, the allocation of donor livers continues to be an area of debate not only for primary liver transplantation but also for orthotopic liver retransplantation, which has inherently different medical, ethical, and economic considerations. Specifically, the opportunity cost of liver retransplantation is that it denies an already scarce resource to first-time liver transplant candidates. More broadly, the economic costs of liver retransplantation may have substantial downstream effects in a constrained healthcare system as it costs over twice that of primary transplantation [8, 9]. Given this, it is critical to re-evaluate the role of liver retransplantation over time as indications and outcomes have likely changed with advances in our understanding of donor selection and recipient management.

To date, liver retransplantation has yet to be studied broadly in Canada. Thus, this investigation aims to assess the incidence, post‐transplant outcomes, and risk factors in recipients of liver retransplantation in Canada. Furthermore, with this nationwide study group, we aim to conduct a subgroup analysis of the characteristics and outcomes of those undergoing more than two liver transplants (i.e., repeat retransplantation (RRT)).

## 2. Materials and Methods

Our study population included all adult (≥18 years) liver retransplant recipients and their matched donors registered on the Canadian Organ Replacement Register (CORR) from 1^st^ January 2000 to 31^st^ December 2018. CORR is a national registry maintained by the Canadian Institute for Health Information (CIHI) that collects information on the majority of Canadian liver transplants but does not include those performed in Quebec. Distinction between different centers are not available in this registry. For this study, simultaneous multiorgan transplants were excluded. The study was approved by CIHI and the research ethics board of University Health Network, Toronto, Canada (REB#19-5835).

Patient-level transplant data included information on liver recipients at the time of LT (age, gender, weight, height, blood type, medical status, model for end-stage liver disease (MELD) score, creatinine, serum bilirubin, international normalized ratio (INR), HCV status, liver disease diagnosis, and cytomegalovirus (CMV)), matched donors (age, gender, weight, height, ethnicity, blood type, cause of death, donor type, HCV status, and CMV status), transplant-specific variables (cold ischemic times), and distance from the donor procurement facility to the transplant facility. Furthermore, post‐transplant outcome data including graft failure, death, reason for death and graft failure, and date of last follow-up were collected. Medical status of a patient was defined as Status 1, patients at home; Status 1T, patients with tumors; Status 2, hospitalized patients; Status 3, patients hospitalized in intensive care unit (ICU); Status 3F, patients with fulminant failure in ICU; Status 4, patients in ICU with intubation/ventilation; and Status 4F, patients with fulminant failure in ICU with intubation/ventilation.

Trends in the number and proportion of yearly retransplantations were assessed with a linear regression least-squared model. A descriptive analysis was performed of all patients receiving retransplantation and stratified by those receiving second, third, and fourth grafts. Descriptive data for continuous variables were expressed as means with standard deviation if the distribution was normal and median with interquartile range (IQR) for non‐normal distribution. Categorical variables were expressed as numbers and percentages.

Given that a patient may die with a functioning graft, the cumulative incidence of graft failure was assessed with a competing risk analysis and compared with the Gray test with death as a competing event [10]. Patient survival was estimated using Kaplan–Meier analysis and compared using the log-rank test. Graft failure and patient survival were assessed at 1-, 3-, 5-, and 10-year time points. Patient survival and graft failure both were calculated from their most recent LT. Retransplant outcome according to acuity of the graft/liver failure was assessed by dividing the cohort by the time from previous LT to retransplantation (hyperacute: 0–7 days, acute: 7–30 days, and chronic: >30 days). A sensitivity analysis was performed in patients who survived at least 30 days post‐transplantation to consider the perioperative period.

For comparative purposes, post‐transplant survival and graft failure in those with single (i.e., primary only) liver transplantations, which met the same inclusion criteria as the retransplantation group, were described. Additionally, to investigate the temporal effects on post‐transplant survival and graft failure in LT, especially for patients who received LT for HCV, the transplant patients were divided into two groups (pre-DAA era: 2000–2010 and post-DAA era: 2011–2018) and were compared.

The association between various predictors and graft failure and patient survival was evaluated using the Fine–Gray competing risk model and Cox regression analyses, respectively. The Fine–Gray model fits a proportional subdistribution hazards' regression model and assesses the effects of covariates on the subdistribution hazard ratio of a particular type of failure in a competing risk setting, in this case, graft failure [11]. The proportional hazard assumption was assessed, and if the assumption was not met, a time-dependent analysis was conducted using the appropriate time period. Multivariable analyses were performed using backward stepwise regression and using all variables with *p* < 0.15 on univariate analysis. Statistical significance was defined as a value of *p* < 0.05. All statistical analyses were performed using IBM SPSS Statistics for Windows, version 26.0 (IBM Corp., Armonk, NY, USA), R (R Core Team (2019) R: A language and environment for statistical computing), and Statistical Analysis System (SAS) version 9.4 (SAS Institute, Cary, NC).

## 3. Results

**Figure 1:**
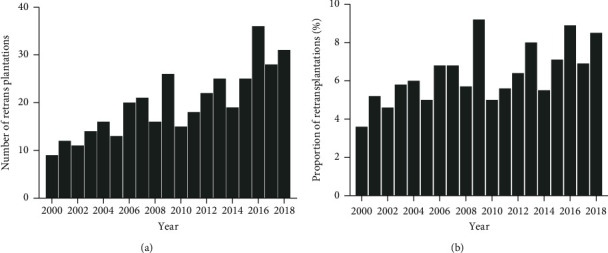
Number and proportion of retransplantations by year from 2000 to 2018.

Over the 18-year study period, 5,805 patients underwent liver transplantation in Canada, with 377 (6.5%) having retransplantations. Of these, 340 (90.2%) received a second graft, 34 (9.0%) a third graft, and 3 (0.8%) a fourth graft. Retransplantation numbers significantly increased over the study period, in which the yearly increase in retransplantations was 1.1 (95% CI: 0.8–1.4, *p* < 0.001), as shown in Figure 1(a). There were nine retransplantations in 2000; meanwhile, 31 retransplantations were performed in 2018. The proportion of retransplantations also increased over the study period with a yearly increase of 0.2% (95% CI: 0.1–0.3, *p*=0.002) (Figure 1(b)). The median follow-up was 3.0 years (IQR: 0.5–8.4). Retransplantation recipient and donor characteristics are summarized in Tables 1 and 2. Notably, autoimmune liver diseases and HCV were the most common underlying liver diagnoses (102 (27.1%) and 64 (17.0%), respectively). Almost a third of the recipients were in the ICU and intubated (Status 4 and 4F) preoperatively. The majority of donors were after declaration of brain death (336, 89.1%). The median distance to the donor procurement facility was 172.7 (IQR: 2.1–21.6) kilometers, and the median cold ischemia time was 443 (IQR: 327–572) minutes.

With regard to the timing of transplantation, the median time between the initial and the second transplants was 47 (IQR: 10–642) days, the second and the third transplants was 546 (IQR: 43–1,998) days, and the third and the fourth transplants was 805 (IQR: NA) days. Retransplantation occurred within 30 days in 123/273 (45.1%) patients in the second transplant group and 5/28 (17.9%) patients in the RRT group.

**Figure 2:**
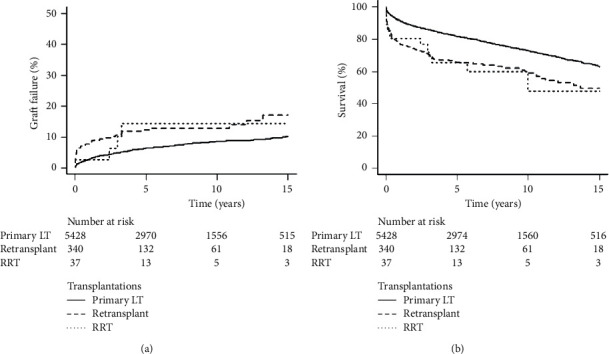
Incidence of graft failure (a) and overall survival (b) by the number of transplantations (LT: liver transplantation; RRT: repeat retransplantation).

In all patients with a retransplantation, the rate of graft failure was 7.9%, 12.5%, and 13.0% and patient survival was 77.1%, 65.6%, and 60.0% at 1, 5, and 10 years, respectively (Table 3 and Figure S1). Causes of graft failure and death in the retransplantation group are presented in Table S1. In contrast to recipients receiving their first graft transplant, the retransplantation group had a significantly higher incidence of graft failure (*p* < 0.001) and lower overall survival (*p* < 0.001) at 1, 5, and 10 years, respectively (Table 3 and Figure S1). For patients who survived beyond 30 days post‐transplantation, the patient survival differences remained between the primary transplantation group and the retransplantation group (Figure S1). Furthermore, there were no significant differences in graft failure and overall survival between the second transplant group and the RRT group (i.e., those receiving a third or fourth graft) (Figure 2). When the time from previous LT to retransplantation was assessed (0–7 days (*n* = 63, 20.9%), 7–30 days (*n* = 65, 21.6%), and >30 days (*n* = 173, 57.4%)), there were no significant differences in graft survival and overall survival.

**Figure 3:**
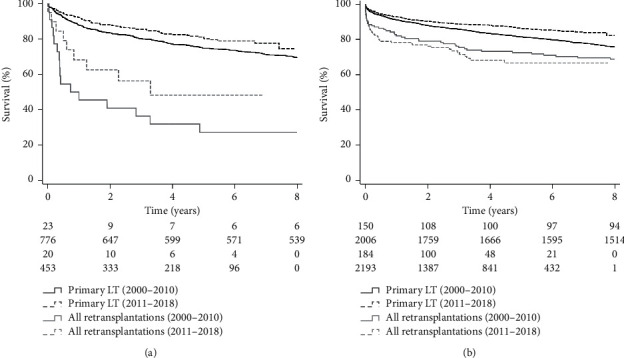
Temporal effects on post‐transplant overall survival (pre-DAA era: 2000–2010 vs. post-DAA era: 2011–2018) (DAA: direct-acting antiviral agents; LT: liver transplantation).

There were temporal effects on post‐transplant overall survival in primary LT from the first era (2000–2010) to the second era (2011–2018) (HCV: *p* < 0.001, other etiologies: *p*=0.03) (Figure 3). For those retransplanted for HCV, post‐transplant overall survival was higher in those from the second era, yet no significant difference was detected, likely due to the small number of patients (*p*=0.15). For other etiologies, there were no statistically significant improvements in post‐transplant survival over time (*p*=0.38).

In the univariate analysis for graft failure, the following parameters were included in the backward stepwise model selection (*p* < 0.15): blood group, weight, donor CMV status, and recipient CMV status. In the overall survival model, the following parameters were included: recipient and donor age, recipient HCV and CMV status, medical status (ventilation), donor CMV status, DCD donor, and facility volume.

In multivariable analysis, donor CMV status was the only factor significantly associated with graft failure (HR = 3.03, 95%CI: 1.28–7.17, *p*=0.01, Table 4). In contrast, recipient age (5-year increase), donor age (5-year increase), and donor CMV status were associated with overall survival (Table 5). Older recipient and donor ages were associated with increased risk for death (recipient: HR = 1.12, 95%CI: 1.03–1.22, *p*=0.011; donor: HR = 1.09, 95%CI: 1.03–1.16, *p*=0.008). The association of donor CMV status with survival was not constant over time and therefore was evaluated as a time-dependent covariate. Donor positivity for CMV was only associated with an increased risk for death following three years after retransplantation (HR = 4.10, 95%CI: 1.50–11.25, *p*=0.006). Whether donor CMV and recipient CMV status were matched had no impact on post‐transplant outcomes. In univariate analysis of post‐transplant survival, the HR for CMV match was 1.13 (95%CI: 0.71–1.80, *p*=0.61) and 1.06 (95%CI: 0.66–1.68, *p*=0.83) in multivariable analysis. For graft survival, CMV match had an HR of 1.13 (95%CI: 0.49–2.57, *p*=0.78) and 0.91 (95%CI: 0.38–2.19, *p*=0.83) in univariate and multivariable analysis, respectively. Furthermore, there were no significant interactions between donor CMV status and recipient CMV status, suggesting that the effect of donor CMV on outcomes is consistent irrespective of recipient CMV status.

Given that the HCV status of recipients was available in a limited number of patients, a secondary model was created, which also included HCV status in the backward stepwise selection, in which it was demonstrated to be associated with overall survival (HR = 1.81, 95%CI: 1.06–3.08, *p*=0.014).

## 4. Discussion

Retransplantation remains the only salvage for patients experiencing graft failure, which would otherwise result in mortality. Yet, scrutiny surrounding the indications and debate on the utility and futility of liver retransplantation persists. This is the largest Canadian study of liver retransplantation, which was noted to be performed in 6.5% of all recipients. Our findings suggest that retransplantation is a valid treatment, with a 5-year graft failure and patient survival of 12.5% and 65.6%, respectively. This is true not only for early graft failure from vascular complications and acute rejection but also for late graft failure from disease recurrence and chronic rejection. RRT, although performed uncommonly, resulted in similar results compared to initial retransplantation in the appropriately selected recipient and should remain an option in the setting of recurrent graft failure.

Worldwide, the incidence of retransplantation ranges from 5.5% to 7% [12–14]. At 6.5%, the overall retransplantation rate in our Canadian cohort was similar. Temporally, we noted that the frequency of retransplantation has steadily increased in Canada from 9 cases in 2000 to 31 cases in 2018, with approximately 0.2% yearly increase in retransplantation rates. Similarly, Australia and New Zealand have also noticed an increase in retransplantation since 2001 [13]. Conversely, this has not been the case in Europe, where an analysis of the European Liver Transplant Registry (ELTR) from 2000–2009 revealed a 5% decrease in the utilization of retransplantation [14].

In our study, compared to initial transplantation, retransplantation was associated with a significantly lower patient and graft survival. Despite this, the results of our multicenter Canadian cohort were consistent with those from other countries, demonstrating an acceptable graft failure rate of 7.9%, 12.5%, and 13% with an overall survival of 77.1%, 65.6%, and 60% at 1, 5, and 10 years, respectively [13–17]. Notably, the most significant drop in survival was observed in the first year post‐transplantation. A study of the ELTR also noted this trend in which half of the deaths and three-quarters of graft failure for European retransplantation occurred within a year [14].

Multiple earlier studies have addressed the interval from previous transplantation as an essential predictor for outcome [3, 15, 17, 18]. These studies state that early retransplantation (<30 days) often has worse outcomes than late retransplantation. In our study, early versus late retransplantation did not have a significant impact on graft failure and overall survival. However, the prevalence of early graft failure differed according to the number of grafts transplanted. Specifically, of recipients with a second graft, 45.1% (*n* = 123) had this within 30 days of their primary transplantation. In contrast, only 17.9% (*n* = 5) of the RRT group had their subsequent transplantation within 30 days.

Given its rarity, minimal data on RRT are available in the current literature. In a single-center review of RRT, the overall incidence was noted to be 2.1% and was performed most commonly for chronic rejection (33%), arterial thrombosis (28%), or primary nonfunction (17%) [19]. In these patients, 90-day mortality for RRT was 18% compared to 15% in those receiving a first retransplant [19]. Notably, of the eight patients receiving a fourth graft, 90-day mortality was 50% [19]. Clinically relevant independent risk factors for 90-day mortality in the RRT group were extrahepatic sepsis and the need for vasopressor support at the time of retransplantation [19]. By performing a nationwide analysis, we were able to perform a subgroup analysis on recipients of RRT. Our RRT rate was 0.6%, similar to the reported incidences of 0.5 to 2.1% from Australia, New Zealand, France, and the USA [13, 19, 20]. Concerning outcomes, Canadian patients with RRT had an acceptable 1-, 5-, and 10-year graft failure and patient survival of 2.7%, 14.3%, and 14.3% and 80.6%, 65.4%, and 59.9%, respectively. This was similar to those receiving a second graft. Thus, RRT remains an option for recurrent graft failure. However, careful selection of patients to achieve the best results remains necessary, given the lack of significant long-term survival benefit in this group of recipients [15, 16, 21].

Over the years, there has been an effort in the literature to identify independent predictors of patient and graft survival after liver retransplantation. Recipient age, creatinine, bilirubin, ventilatory status, MELD, United Network for Organ Sharing (UNOS) status, urgency of retransplantation, cold ischemia time, and causes of graft failure have been noted to be significant variables [16, 18, 22, 23]. Our Canadian cohort study adds to the heterogeneity in the literature as it demonstrated recipient age, donor age, and donor CMV status to be independent predictors of overall survival. Donor age has been confirmed as a predictor of prognosis for retransplantation [21]. Marudanayagam et al. showed that donor age <55 years (OR:1.02, 95%CI: 1.00–1.04, *p*=0.036) correlates with lower mortality along with MELD score <23 (OR:1.103, 95%CI: 1.00–1.06, *p*=0.029) in their single-center series [21]. This was confirmed in our study, in which older recipients and patients who received grafts from an older donor had worse outcomes. CMV is a common viral pathogen that can adversely affect liver transplant recipients and is associated with CMV syndrome, tissue-invasive diseases, an increased predisposition to rejection and mortality, accelerated HCV recurrence, and other opportunistic infections [24]. Although there is a lack of specific evidence regarding the correlation between CMV status and liver retransplantation outcome, its impact can be inferred from studies on primary liver transplants. Donor and recipient CMV-positive status have been shown to adversely affect recipient survival in primary liver transplantation (RR:4.6, 95%CI: 1.9–10.7, *p* < 0.001) [25]. CMV-positive patients who received a liver transplant are also more prone to have graft failure than CMV-negative patients [26]. Our Canadian series has demonstrated its negative impact on graft failure (HR: 3.03, 95%CI: 1.28–7.17, *p*=0.01) and patient survival following 3 years post‐retransplantation (HR: 4.10, 95%CI: 1.50–11.25, *p*=0.006). Contrastingly, CMV match status between the donor and the recipient had no impact on post‐transplantation outcomes in addition to donor CMV status, as shown in our multivariable analysis of post‐transplant outcomes.

In the current study, HCV was the second most common underlying liver diagnosis (64, 16.9%) and associated with increased patient mortality (HR: 1.81, 95%CI: 1.06–3.08, *p*=0.014). Historically, HCV positivity as an indicator for worse outcomes in retransplantation has been controversial. Rosen et al. identified HCV infection as an independent risk factor for death after retransplantation (RR: 1.36, *p*=0.0038) [27]. Watt et al. showed through analysis of UNOS data on 2,129 retransplantation patients that HCV patients had no different survival compared to retransplantation for HBV and autoimmune hepatitis [28]. This was further supported by this group's subsequent study, where 1- and 3-year survival rates after retransplantation were not significantly different for HCV and non‐HCV groups (69 vs. 73% and 49 vs. 55%, respectively) [29]. These data are changing with the increased use of DAA (after 2010) to manage this underlying etiology of end-stage liver disease. This temporal effect was observed in our post‐transplant outcomes in primary LT from the first era (2000–2010) to the second era (2011–2018). Primary LT patients for HCV in the second era had superior overall survival than patients in the first era (*p* < 0.001). However, for those retransplanted for HCV, the difference in post‐transplant survival was only observed without statistical significance likely due to the small number of patients (*p*=0.15).

This study is limited in that we do not address death on the waitlist in patients listed for retransplantation. Additionally, selection bias cannot be accounted for given the multicenter design of this study and the lack of insight into donor and recipient matching considerations. Furthermore, inherent to the dataset, the analysis was limited by each center's accuracy and completeness of data entered into the national registry.

## 5. Conclusion

This report represents the largest analysis of liver retransplantation in Canada. It demonstrates that this is a feasible and safe treatment for early and late liver graft failure. Furthermore, even in patients requiring RRT, with careful selection, similar outcomes to initial retransplantation can be achieved.

## Figures and Tables

**Table 1:** Liver retransplant recipient characteristics.

	Overall	2 transplants	3 transplants	4 transplants
	*N* = 377	*N* = 340 (90.2%)	*N* = 34 (9.0%)	*N* = 3 (0.8%)
Age at LT, years, median (IQR)	52 (42–59)	52 (44–59)	43 (33–52)	34
Male, number (%)	225 (59.7)	203 (59.7)	19 (55.9)	3 (100.0)
Time from previous transplant, days, median (IQR)	5 (10–809)	47 (10–642)	546 (43–1998)	805
Unknown (*n*) = 76 (20.2%)				
Creatinine at LT, *μ*mol/L, median (IQR)	104 (82–183)	103 (80–186)	113 (97–179)	117
Unknown (*n*) = 150 (39.8%)				
Serum bilirubin at LT, *μ*mol/L, median (IQR)	135.5 (52.8–409.5)	135.5 (53.8–414.5)	117.0 (32.0–370.0)	277.0
Unknown (*n*) = 151 (40.1%)				
INR at LT, median (IQR)	1.6 (1.3–2.3)	1.6 (1.3–2.4)	1.6 (1.2–1.8)	1.4
Unknown (*n*) = 153 (40.6%)				
Blood type, number (%)				
A	172 (45.6)	153 (45.0)	17 (50.0)	2 (66.7)
AB	17 (4.5)	15 (4.4)	1 (2.9)	1 (33.3)
B	58 (15.4)	56 (16.5)	2 (5.9)	0 (0.0)
O	129 (34.2)	116 (34.1)	13 (38.2)	0 (0.0)
Unknown	1 (0.3)	0 (0.0)	1 (2.9)	0 (0.0)
Liver diagnosis, number (%)				
AIH/PSC/PBC	102 (27.1)	91 (26.8)	10 (29.4)	1 (33.3)
Alcoholic cirrhosis	20 (5.3)	20 (5.9)	0 (0.0)	0 (0.0)
Hepatocellular carcinoma	21 (5.6)	18 (5.3)	3 (8.8)	0 (0.0)
HBV	21 (5.6)	20 (5.9)	1 (2.9)	0 (0.0)
HCV	64 (17.0)	61 (17.9)	3 (8.8)	0 (0.0)
NASH	10 (2.7)	10 (2.9)	0 (0.0)	0 (0.0)
Cryptogenic	62 (16.4)	51 (15.0)	10 (29.4)	1 (33.3)
Other	77 (20.4)	69 (20.3)	7 (20.6)	1 (33.3)
Previous organ failure cause, number (%)				
Primary nonfunction	31 (8.2)	29 (8.5)	2 (5.9)	0 (0.0)
Portal vein thrombosis	4 (1.1)	4 (1.2)	0 (0.0)	0 (0.0)
Hepatic vein thrombosis	18 (4.8)	16 (4.7)	2 (5.9)	0 (0.0)
Hepatic artery thrombosis	38 (10.1)	36 (10.6)	1 (2.9)	1 (33.3)
Acute rejection	4 (1.1)	4 (1.2)	0 (0.0)	0 (0.0)
Chronic rejection	11 (2.9)	11 (3.2)	0 (0.0)	0 (0.0)
Recurrence of original disease	23 (6.1)	21 (6.2)	2 (5.9)	0 (0.0)
Other	19 (5.0)	18 (5.3)	1 (2.9)	0 (0.0)
Unknown/uncertain	229 (60.8)	201 (59.1)	26 (76.5)	2 (66.7)
BMI, kg/m^2^, median (IQR)	24.9 (21.9–28.7)	25.2 (22.2–29.0)	23.9 (20.1–25.9)	24.2
Unknown (*n*) = 27 (7.2%)				
MELD score at LT, median (IQR)	24 (17–31)	24 (17–32)	23 (16–27)	23
Unknown (*n*) = 154 (40.8%)				
Medical status, number (%)				
At home (1, 1T)	74 (19.6)	65 (19.1)	8 (23.5)	1 (33.3)
Hospitalized (2)	75 (19.9)	65 (19.1)	10 (29.4)	0 (0.0)
Hospitalized/ICU (3, 3F)	44 (11.7)	40 (11.8)	3 (8.8)	1 (33.3)
ICU/ventilated (4, 4F)	124 (32.9)	117 (34.4)	6 (17.7)	1 (33.3)
Unknown	60 (16.0)	53 (15.6)	7 (20.6)	0 (0.0)
HCV positive, number (%)	54 (14.3)	53 (15.6)	1 (2.9)	0 (0.0)
Unknown (*n*) = 139 (36.9%)				
CMV positive, number (%)	182 (48.3)	165 (48.5)	17 (50.0)	0 (0.0)
Unknown (*n*) = 116 (30.8%)				
CIT, minutes, median (IQR)	443 (327–572)	443 (329–572)	456 (289–590)	474
Unknown (*n*) = 87 (23.1%)				

LT, liver transplantation; IQR, interquartile range; INR, international normalized ratio; AIH, autoimmune hepatitis; PSC, primary sclerosing cholangitis; PBC, primary biliary cholangitis; HBV, hepatitis B virus; HCV, hepatitis C virus; NASH, nonalcoholic steatohepatitis; BMI, body mass index; MELD, model for end-stage liver disease; ICU, intensive care unit; CMV, cytomegalovirus; CIT, cold ischemia time.

**Table 2:** Liver retransplant donor characteristics.

	Overall	2 transplants	3 transplants	4 transplants
	*N* = 377	*N* = 340	*N* = 34	*N* = 3
Age at death, years, median (IQR)	41 (24–54)	41 (23–54)	43 (29–51)	44
Unknown (*n*) = 33 (8.8%)				
BMI, kg/m^2^, median (IQR)	24.8 (22.0–27.7)	25.0 (22.0–27.5)	23.7 (22.0–28.2)	30.1
Unknown (*n*) = 52 (13.8%)				
Male sex, number (%)	187 (49.6)	171 (50.3)	15 (44.1)	1 (33.3)
Cause of death, number (%)				
Anoxia	63 (16.7)	57 (16.8)	6 (17.6)	0 (0.0)
Cerebrovascular accident	138 (33.6)	121 (35.6)	15 (44.1)	2 (66.7)
Trauma	101 (26.9)	94 (27.7)	6 (17.6)	1 (33.3)
Overdose	9 (2.4)	7 (2.1)	2 (5.9)	0 (0.0)
Unknown	49 (13.0)	47 (13.8)	2 (5.9)	0 (0.0)
Other	17 (4.6)	14 (4.1)	3 (8.8)	0 (0.0)
Ethnicity, number (%)				
Caucasian	219 (58.1)	194 (57.1)	24 (70.6)	1 (33.3)
Asian	13 (3.4)	11 (3.2)	1 (2.9)	1 (33.3)
Black	7 (1.9)	6 (1.8)	1 (2.9)	0 (0.0)
Other/unknown	138 (36.6)	129 (37.9)	8 (23.6)	1 (33.3)
Donor type, number (%)				
DCD	8 (2.1)	7 (2.1)	1 (2.9)	0 (0.0)
NDD	336 (89.1)	301 (88.5)	32 (94.1)	0 (0.0)
Unknown	33 (8.8)	32 (9.4)	1 (2.9)	3 (100.0)
CMV, number (%)	153 (40.6)	137 (40.3)	14 (41.2)	2 (66.7)
Unknown (*n*) = 75 (19.9%)				
Hepatitis C, number (%)	4 (1.1)	3 (0.9)	1 (2.9)	0 (0.0)
Unknown (*n*) = 66 (17.5%)				
Blood type, number (%)				
A	124 (32.9)	109 (32.1)	14 (41.2)	1 (33.3)
AB	11 (2.9)	9 (2.6)	1 (2.9)	1 (33.3)
B	41 (10.9)	39 (11.5)	2 (5.9)	0 (0.0)
O	167 (44.3)	150 (44.1)	16 (47.1)	1 (33.3)
Unknown	34 (9.0)	33 (9.7)	1 (2.9)	0 (0.0)
Distance from donor harvest to the facility of LT, km, median (IQR)	172.7 (2.1–621.6)	169.6 (2.0–672.3)	273.8 (2.2–520.4)	1,398.4
Unknown (*n*) = 84 (22.3%)				

IQR, interquartile range; BMI, body mass index; DCD, donor after cardiac death; NDD, neurological determination of death; CMV, cytomegalovirus; LT, liver transplantation.

**Table 3:** Graft failure incidence and patient survival at various time points according to the number of transplantations.

	Graft failure incidence (%) (95%CI)			
	1 year	3 year	5 year	10 year
Primary LT	2.9 (2.5–3.4)	5.0 (4.4–5.6)	6.4 (5.8–7.2)	8.6 (7.8–9.5)
Retransplantation	8.4 (5.7–11.7)	11.0 (7.8–14.8)	12.3 (8.9–16.3)	12.8 (9.3–17.0)
RRT	2.7 (0.2–12.3)	10.2 (2.4–24.7)	14.3 (4.3–30.2)	14.3 (4.3–30.2)
All retransplantations	7.9 (5.4–10.9)	10.9 (7.9–14.5)	12.5 (9.2–16.4)	13.0 (9.6–16.9)

	Patient survival (%) (95%CI)			
	1 year	3 year	5 year	10 year
Primary LT	91.4 (90.6–92.1)	86.0 (85.0–86.9)	81.8 (80.7–82.9)	72.9 (71.4–74.3)
Retransplantation	76.8 (71.8–81.0)	70.5 (65.0–75.3)	65.6 (59.8–70.8)	60.1 (53.5–66.1)
RRT	80.6 (63.6–90.3)	69.5 (50.1–82.5)	65.4 (45.4–79.5)	59.9 (39.0–75.7)
All retransplantations	77.1 (72.4–81.1)	70.4 (65.2–74.9)	65.6 (60.1–70.5)	60.0 (53.7–65.7)

LT, liver transplantation; RRT, repeat retransplantation.

**Table 4:** Multivariable analysis after backward stepwise selection of predictors for graft failure in retransplantation.

	Graft failure (*n* = 283)		
	HR	95% CI	*p* value
Recipient weight	0.98	0.96–1.00	0.073
Donor CMV-positive	3.03	1.28–7.17	0.01

**Table 5:** Multivariable analysis after backward stepwise selection of predictors for patient survival in retransplantation.

	Patient survival (*n* = 196)			Patient survival (*n* = 302)		
	Including HCV status			Excluding HCV status		
	HR	95% CI	*p* value	HR	95% CI	*p* value
Recipient age (5-year increase)	—	—	—	1.12	1.03–1.22	0.011
Recipient HCV-positive	1.81	1.06–3.08	0.014			
Donor age (5-year increase)	—	—	—	1.09	1.03–1.16	0.008
Donor CMV-positive (≤3 years)	1.29	0.73–2.28	0.37	1.26	0.80–1.97	0.32
Donor CMV-positive (>3 years)	6.59	1.85–23.54	0.004	4.10	1.50–11.25	0.006

## Data Availability

Our study population included all adult (≥18 years) liver retransplant recipients and their matched donors registered on the Canadian Organ Replacement Register (CORR) from 1st January 2000 to 31st December 2018. CORR and the centers participating in the CORR are the source of the data used herein; they have not verified and are not responsible for the statistical validity of the data analysis of the conclusions derived by the authors.

## Abbreviations

CI: Confidence interval
CIHI: Canadian Institute for Health Information
CORR: Canadian Organ Replacement Register
CMV: Cytomegalovirus
ELTR: European Liver Transplant Registry
HCV: Hepatitis C virus
HR: Hazard ratio
ICU: Intensive care unit
INR: International normalized ratio
IQR: Interquartile range
LT: Liver transplantation
MELD: Model for end-stage liver disease
NA: Not applicable
OR: Odds ratio
RR: Relative ratio
RRT: Repeat retransplantation
UNOS: United Network for Organ Sharing.

## Supplementary Materials

Supplementary Materials
*Table S1*. Cause of graft failure and death in patients with retransplantation. *Supplementary Figure 1*. Incidence of graft failure and patient survival from the time of transplantation (A) and from 30 days after transplantation (B), according to retransplantation status (LT: liver transplantation).
