# Supplementary material for: Outcomes of Adult Liver Retransplantation: A Canadian National Database Analysis
Source: Can J Gastroenterol Hepatol. 2022 Mar 22;2022:9932631. doi: 10.1155/2022/9932631 (PMC8964213; doi:10.1155/2022/9932631)
Supplement: Supplementary Materials — Table S1. Cause of graft failure and death in patients with retransplantation. Supplementary Figure 1. Incidence of graft failure and patient survival from the time of transplantation (A) and from 30 days after transplantation (B), according to retransplantation status (LT: liver transplantation). [file 9932631.f1.docx]

**Supplementary**

**Table S1.** Cause of graft failure and death in patients with retransplantation

|  | **Overall**  **N=377** | **2 Transplants**  **N=340** | **3 transplants**  **N=34** | **4 transplants**  **N=3** |
| --- | --- | --- | --- | --- |
| Graft Failure Cause, number (%)  Total  Primary Non-Function  Hepatic Vein Thrombosis  Hepatic Artery Thrombosis  Acute Rejection  Chronic Rejection  Recurrence of Original Disease  Other  Unknown/Uncertain | 46  2 (4.3)  1 (2.2)  1 (2.2)  1 (2.2)  3 (6.5)  8 (17.4)  10 (21.7)  20 (43.5) | 42  2 (4.8)  1 (2.4)  1 (2.4)  1 (2.4)  3 (7.1)  6 (14.3)  10 (23.8)  18 (42.9) | 4  0 (0.0)  0 (0.0)  0 (0.0)  0 (0.0)  0 (0.0)  2 (50.0)  0 (0.0)  2 (50.0) | 0  0 (0.0)  0 (0.0)  0 (0.0)  0 (0.0)  0 (0.0)  0 (0.0)  0 (0.0)  0 (0.0) |
| Cause of Death, number (%)  Total  Cardiac  Cerebrovascular Accident  Septicaemia/sepsis  Liver Failure  Malignancy  Multi System Failure  Other/Uncertain | 134  6 (4.4)  6 (4.5)  21 (15.7)  17 (12.7)  11 (8.2)  26 (19.4)  47 (35.1) | 121  6 (4.9)  6 (5.0)  21 (17.4)  16 (13.2)  8 (6.6)  25 (20.7)  39 (27.3) | 12  0 (0.0)  0 (0.0)  0 (0.0)  1 (8.3)  3 (25.0)  1 (8.3)  7 (50.0) | 1  0 (0.0)  0 (0.0)  0 (0.0)  0 (0.0)  0 (0.0)  0 (0.0)  1 (100.0) |


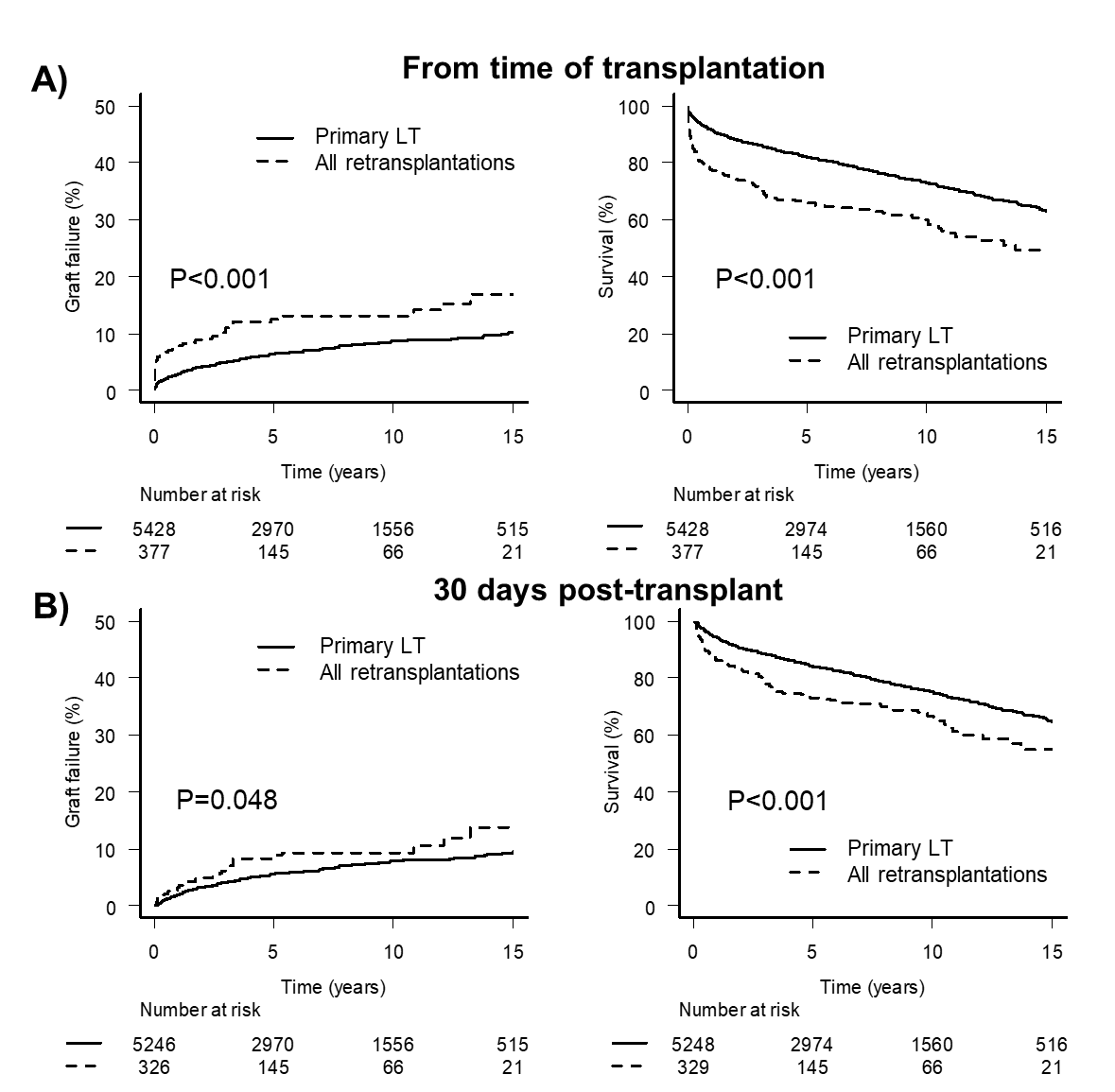


**Supplementary Figure 1.** Incidence of graft failure and patient survival from time of transplantation (A), and from 30 days after transplant (B), according to retransplantation status (LT: Liver transplant)
